# Childhood maltreatment and adolescent non-suicidal self-injury: cross-sectional conditional process and network analyses of depressive symptoms and physical activity

**DOI:** 10.3389/fpsyt.2026.1899267

**Published:** 2026-09-15

**Authors:** Wanchun Xue, Jianhua Zhang, Chang Hong

**Affiliations:** 1 Guangzhou Sport University, Guangzhou, Guangdong, China; 2 School of Physical Education and Health, Huaihua University, Huaihua, China

**Keywords:** adolescents, childhood maltreatment, conditional process analysis, depressive symptoms, network analysis, non-suicidal self-injury, physical activity

## Abstract

**Background:**

Childhood maltreatment is associated with adolescent non-suicidal self-injury (NSSI), but the roles of depressive symptoms and physical activity remain unclear. We examined these relationships using variable-level and network approaches.

**Methods:**

This cross-sectional study included 2,354 adolescents recruited by convenience sampling from 30 secondary schools in six Chinese provinces. The 12-item behavior dimension of the Adolescent Non-Suicidal Self-Injury Assessment Questionnaire was the primary outcome. Covariate-adjusted indirect-effect and conditional process analyses used HC3 standard errors and 5,000 bootstrap samples; an 18-node regularized partial-correlation network was also estimated.

**Results:**

Childhood maltreatment correlated positively with depressive symptoms (r = .365) and NSSI behavior (r = .366), while depressive symptoms correlated positively with NSSI behavior (r = .531; all p < .001). Physical activity was negatively associated with depressive symptoms and NSSI behavior. Depressive symptoms showed a significant cross-sectional indirect association between childhood maltreatment and NSSI behavior (effect = .084, 95% bootstrap CI [.072, .098]). The depressive symptoms × physical activity interaction was statistically significant but very small (B = −.015, 95% CI [−.021, −.010]; ΔR^2^ = .013; f^2^ = .019), indicating limited practical significance despite the large sample size. Results remained stable after excluding the PHQ-9 self-harm item. Network analysis identified emotional neglect, sleep problems, and physical neglect as prominent positive bridge nodes, while the single NSSI node showed high cross-community connectivity; physical activity showed only weak cross-community connections after accounting for the remaining nodes.

**Conclusions:**

Depressive symptoms statistically accounted for part of the childhood maltreatment–NSSI association. Although physical activity significantly interacted with depressive symptoms, the interaction explained only 1.3% of additional variance and had limited practical significance. The weak physical activity connections in the network further suggested little unique symptom-level association after shared variance was controlled. Because the data were cross-sectional, temporal mediation and causal inferences cannot be established, and clinical benefit cannot be inferred.

## 1 Introduction

Non-suicidal self-injury (NSSI) refers to the deliberate and direct destruction of one’s own body tissue without explicit suicidal intent (1–3). As a relatively common but highly consequential high-risk behavior among adolescents, NSSI is associated with depression, anxiety, emotion-regulation difficulties, suicidal ideation, and an increased risk of subsequent suicidal behavior (4–8). Adolescent NSSI should therefore not be regarded as an isolated individual behavior. Rather, it is a complex developmental and public health concern embedded within the interacting influences of family relationships, school contexts, peer interactions, academic pressure, emotional distress, and available coping resources (9–13).

Epidemiological evidence demonstrates that childhood maltreatment, depressive symptoms, and NSSI are common among Chinese children and adolescents. A meta-analysis of Chinese primary- and middle-school students reported pooled prevalence estimates of 20% for physical abuse, 30% for emotional abuse, 12% for sexual abuse, 47% for physical neglect, and 44% for emotional neglect (14). A recent meta-analysis involving 439 studies and 1,497,524 Chinese children and adolescents estimated the pooled prevalence of depressive symptoms at 26.17% (15). Another meta-analysis reported a pooled NSSI prevalence of 22.37% among Chinese middle- and high-school students (16). Internationally, a large school-based study across 11 US states reported a 12-month NSSI prevalence of 17.6% (17). The 2023 US Youth Risk Behavior Survey further showed that 39.7% of high-school students experienced persistent sadness or hopelessness and 28.5% reported poor mental health (18). These estimates are not directly comparable because the studies differed in sampling methods, assessment instruments, case definitions, and recall periods. Nevertheless, they collectively demonstrate that childhood adversity, emotional distress, and self-injurious behavior constitute substantial concerns across cultural contexts.

Existing research suggests that adolescent NSSI may represent a maladaptive coping response associated with early adverse experiences, proximal psychological distress, and insufficient protective resources (19). From a broader developmental perspective, understanding why some adolescents report NSSI requires examining both the distal experiences associated with increased vulnerability and the modifiable resources that may weaken the association between psychological distress and self-injurious behavior.

The conceptual framework of the present study integrates developmental psychopathology with the functional model of NSSI. Developmental psychopathology proposes that adolescent psychological and behavioral outcomes reflect the combined influence of distal developmental risks, proximal psychological processes, and protective resources (5, 20–22). Within this framework, childhood maltreatment represents a distal developmental risk, depressive symptoms represent a proximal psychological correlate, and physical activity represents a potentially relevant behavioral correlate. The functional model of NSSI further proposes that self-injury may be maintained because it temporarily reduces negative affect, interrupts emotional numbness, communicates distress, or restores a perceived sense of control.

Among the potential distal risk factors, childhood maltreatment has been consistently associated with adolescent NSSI (23–25). Childhood maltreatment generally includes emotional abuse, physical abuse, sexual abuse, emotional neglect, and physical neglect (26–28). Repeated exposure to emotional invalidation, threat, neglect, or inadequate caregiving may be associated with disruptions in attachment security, self-evaluation, perceived social support, stress responsivity, and emotion regulation (29–33). Previous studies have also found that childhood maltreatment is associated with more frequent or severe self-injurious behavior (25, 34, 35). Accordingly, the present study proposed Hypothesis 1: childhood maltreatment is positively correlated with adolescent NSSI.

Childhood maltreatment may also be indirectly associated with NSSI through depressive symptoms (29, 36–38). Depressive symptoms include low mood, loss of interest, sleep disturbance, fatigue, appetite change, negative self-evaluation, concentration difficulties, and hopelessness (39–41). Adolescents exposed to maltreatment may develop greater vulnerability to depressive symptoms through negative self-beliefs, impaired emotion regulation, heightened sensitivity to stress, and reduced perceived support (42–45). Accordingly, the present study proposed Hypothesis 2: childhood maltreatment is positively correlated with depressive symptoms.

Depressive symptoms may, in turn, be associated with greater NSSI frequency. According to the functional model of NSSI, self-injury may provide short-term relief from intense negative emotions or help interrupt emotional numbness (46–48). Adolescents experiencing persistent sadness, hopelessness, self-blame, or perceived lack of control may therefore be more likely to report NSSI when more adaptive coping strategies are unavailable (49–53). Previous studies have consistently identified positive associations between depressive symptoms and adolescent NSSI (7, 50, 54). Based on this evidence, the present study proposed Hypothesis 3: depressive symptoms are positively correlated with adolescent NSSI and statistically account for a significant indirect association between childhood maltreatment and NSSI.

Although depressive symptoms are strongly associated with NSSI, not all adolescents experiencing depressive symptoms engage in self-injury (55–57). Physical activity may be associated with variation in the strength of the depression–NSSI relationship. From a cognitive-behavioral perspective, physical activity may promote behavioral activation, reduce withdrawal and rumination, enhance self-efficacy, and provide opportunities for social interaction and perceived competence (57, 58). These processes may provide alternative ways of responding to sadness, hopelessness, tension, and emotional numbness. From a biological perspective, physical activity may also support stress regulation and endogenous reward and pain-regulation systems associated with affect regulation (59–62).

These mechanisms provide a rationale for placing physical activity on the association between depressive symptoms and NSSI rather than on the association between childhood maltreatment and depressive symptoms. Physical activity cannot alter previous maltreatment exposure, but it may influence how adolescents respond to current depressive distress and whether they rely on NSSI as a short-term emotion-regulation strategy. Accordingly, the present study proposed Hypothesis 4: physical activity moderates the positive correlation between depressive symptoms and adolescent NSSI, such that this positive correlation is weaker at higher levels of physical activity.

Although previous studies have examined associations among childhood maltreatment, depressive symptoms, physical activity, and NSSI, most have focused on pairwise relationships or total scale scores (63–65). Variable-level analyses are useful for evaluating overall associations but may conceal differences among specific maltreatment dimensions and depressive symptoms (66–68). For example, emotional neglect may occupy a different position from physical abuse, and sleep disturbance may be connected with NSSI differently from other depressive symptoms. Network analysis provides a complementary approach for identifying conditional associations and bridge nodes among theoretically distinct psychological domains.

To address these issues while retaining the original variable-level framework, the present study combined conditional-process analysis with exploratory network analysis. The variable-level analysis examined the cross-sectional indirect association between childhood maltreatment and NSSI through depressive symptoms and whether physical activity moderated the depression–NSSI association. Network analysis was used as a supplementary approach to identify specific maltreatment dimensions and depressive symptoms that connected the study domains. Because all variables were measured concurrently, the indirect-effect, moderation, and network results were interpreted as cross-sectional statistical associations rather than evidence of temporal or causal mechanisms.

Accordingly, the present study aimed to examine the correlations among childhood maltreatment, depressive symptoms, physical activity, and adolescent NSSI; test the indirect statistical association between childhood maltreatment and NSSI through depressive symptoms; determine whether physical activity moderated the association between depressive symptoms and NSSI; and identify bridge nodes using network analysis. The following hypotheses were proposed:

H1: Childhood maltreatment is positively correlated with adolescent NSSI.
H2: Childhood maltreatment is positively correlated with depressive symptoms.
H3: Depressive symptoms are positively correlated with adolescent NSSI and statistically account for a significant indirect association between childhood maltreatment and NSSI.
H4: Physical activity moderates the positive correlation between depressive symptoms and adolescent NSSI, such that this correlation is weaker at higher levels of physical activity.

In addition to these hypothesis-driven analyses, the study exploratorily examined whether physical activity moderated the direct association between childhood maltreatment and NSSI. Network analysis was conducted as a supplementary exploratory approach. The proposed conditional-process model is presented in **Figure 1**.

**Figure 1:**
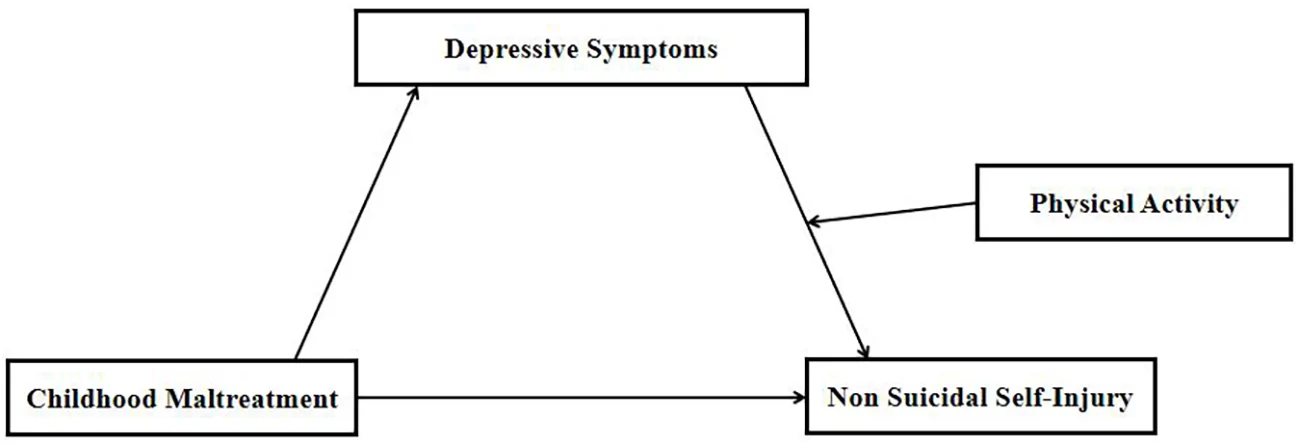
Hypothesized conditional-process model.

## 2 Methods

### 2.1 Participants

This cross-sectional study employed convenience sampling to recruit adolescents from secondary schools in six provinces of China: Sichuan, Hubei, Guangxi, Gansu, Hebei, and Fujian. Five schools were recruited from each province, resulting in a total of 30 participating schools. The participating schools included both urban and rural schools and comprised both key and ordinary schools. Convenience sampling was adopted because access to the schools depended on the cooperation of local educational authorities and participating schools and enabled class-based recruitment across geographically dispersed regions.

The fieldwork period, including recruitment, consent procedures, and questionnaire administration, extended from April 18 to May 17, 2026. Following ethics approval, study information sheets and written informed-consent forms were distributed to parents or legal guardians through the participating schools. Completed consent forms were returned to the schools before the scheduled survey session, and questionnaire administration was arranged separately for each participating school or class after parental or guardian consent had been confirmed. On the day of data collection, adolescents received an age-appropriate explanation of the study, including its purpose, procedures, confidentiality protections, voluntary nature, and right to withdraw without penalty, and provided assent before completing the questionnaire. Only students for whom both written parental or guardian consent and adolescent assent had been obtained participated in the study. Paper-and-pencil questionnaires were subsequently administered on a class-by-class basis with the assistance of homeroom teachers who had received standardized instructions.

A total of 2,500 questionnaires were distributed. Questionnaires with substantial incompleteness or evident invalid-response patterns that prevented reliable scoring were excluded. Ultimately, 2,354 valid questionnaires were retained, yielding an effective response rate of 94.16%. The final sample consisted of 1,098 boys (46.64%) and 1,256 girls (53.36%), with a mean age of 14.60 years (SD = 1.38). Of the participants, 422 (17.93%) reported an urban family residence and 1,932 (82.07%) reported a rural family residence.

The demographic questionnaire collected information on grade, sex, ethnicity, family residence, left-behind status, boarding status, only-child status, paternal and maternal educational level, monthly household income, and family type. These variables were considered potential demographic and socioeconomic confounders because they may be associated with childhood maltreatment, depressive symptoms, physical activity, and non-suicidal self-injury. Detailed demographic and socioeconomic characteristics of the sample are presented in **Supplementary Table 1**.

The study protocol was approved by the Ethics Committee of Jishou University on April 17, 2026 (Approval No. JSDX-2026-0077). All procedures were conducted in accordance with the ethical principles of the Declaration of Helsinki.

### 2.2 Measures

#### 2.2.1 Childhood maltreatment

Childhood maltreatment was assessed using the Childhood Trauma Questionnaire–Short Form (CTQ-SF) (69). The CTQ-SF contains 28 items, of which 25 items assess five dimensions of childhood maltreatment: emotional abuse, physical abuse, sexual abuse, emotional neglect, and physical neglect. The remaining three items are minimization/denial validity items and were not included in the childhood maltreatment total score.

Each item is rated on a five-point Likert scale ranging from 1 (“never true”) to 5 (“very often true”). The positively worded items were reverse-coded before scoring. The scores of the 25 clinical items were summed to produce a total childhood maltreatment score ranging from 25 to 125, with higher scores indicating greater exposure to childhood maltreatment. Childhood maltreatment was treated as a continuous variable, and no diagnostic cutoff was used in the primary analyses. In the present study, Cronbach’s α was.933 for the 25 clinical items. The Cronbach’s α coefficients of the five subscales ranged from.682 to.753, indicating acceptable internal consistency considering the small number of items in each subscale (70).

#### 2.2.2 Depressive symptoms

Depressive symptoms during the preceding two weeks were assessed using the nine-item Patient Health Questionnaire (PHQ-9) (71). Each item is rated from 0 (“not at all”) to 3 (“nearly every day”), resulting in a total score ranging from 0 to 27, with higher scores indicating more severe depressive symptoms. The continuous PHQ-9 total score was used in all primary analyses.

Because the ninth item of the PHQ-9 assesses thoughts of death or self-harm and may conceptually overlap with the study outcome, a sensitivity analysis was also conducted using the PHQ-8 score obtained after excluding Item 9. Cronbach’s α was.772 for the PHQ-9 and.725 for the PHQ-8 in the present study.

#### 2.2.3 Adolescent non-suicidal self-injury

Non-suicidal self-injury (NSSI) was assessed using the Adolescent Non-Suicidal Self-Injury Assessment Questionnaire (ANSAQ) (72). The complete questionnaire contains 31 items divided into two dimensions. Items 1–12 assess the occurrence and frequency of different NSSI behaviors, whereas Items 13–31 assess the functions of NSSI.

Only the 12-item behavior dimension was used because the 19-item function dimension assesses motivations underlying NSSI rather than behavioral frequency and was therefore excluded from the outcome score and network analysis.

Participants reported the frequency of each NSSI behavior during the preceding 12 months. The original responses, coded from 1 to 5 in the dataset, were recoded to a 0–4 scale by subtracting one from each item. The 12 recoded items were then summed, yielding an NSSI behavior score ranging from 0 to 48. Higher scores indicate a higher frequency of NSSI behavior rather than greater functional severity. The score was treated as a continuous outcome, and no diagnostic cutoff was applied. Cronbach’s α for the 12-item NSSI behavior dimension was.856 in the present study.

#### 2.2.4 Physical activity

Physical activity during the preceding month was assessed using the Physical Activity Rating Scale-3 (PARS-3) (73). The PARS-3 contains three items assessing exercise intensity, duration per session, and frequency. Each component is rated on a five-point scale. The total physical activity score was calculated using the standard formula: Physical activity score = exercise intensity × (exercise duration − 1) × exercise frequency.

The total score ranges from 0 to 100, with higher scores indicating a greater level of physical activity. The continuous total score was used in the variable-level analyses, and no arbitrary physical activity cutoff was applied. In the network analysis, exercise intensity, duration, and frequency were retained as three separate nodes to examine their potentially distinct relationships with childhood maltreatment and depressive symptoms. Cronbach’s α for the PARS-3 was.805 in the present study.

### 2.3 Statistical analysis

Statistical analyses were conducted using IBM SPSS Statistics 27.0 and R. Before the primary analyses, data were screened for missing values, outliers, and distributional characteristics. There were no missing values for the primary study variables. One participant had missing data for maternal educational level. Available cases were used for descriptive statistics, whereas adjusted regression and conditional process analyses involving maternal education were based on complete cases (N = 2,353). No missing-value imputation was performed. Histograms, Q–Q plots, skewness, and kurtosis were examined to evaluate variable distributions. Because physical activity and NSSI scores showed non-normal and positively skewed distributions, heteroscedasticity-consistent HC3 standard errors and bootstrap confidence intervals were used for the regression-based analyses.

Means, standard deviations, and Pearson correlation coefficients were first calculated for the principal study variables. Grade, sex, ethnicity, family residence, left-behind status, boarding status, only-child status, paternal education, maternal education, monthly household income, and family type were included as covariates in the adjusted regression analyses. Categorical variables were dummy-coded, and “do not know” responses to the household-income item were retained as a separate response category.

To assess potential common method bias, a confirmatory factor analysis (CFA)-based approach was used instead of relying solely on Harman’s single-factor test (74). Fourteen observed indicators were included: the five CTQ-SF subscale scores; three PHQ-9 parcels; three NSSI behavior parcels; and the three PARS-3 components. A theoretically specified four-factor model was compared with a single-factor model. Model fit was evaluated using CFI, TLI, RMSEA, and SRMR (75). A substantially poorer fit of the single-factor model was interpreted as evidence against a dominant common factor.

The indirect association of childhood maltreatment with NSSI through depressive symptoms was examined using the PROCESS macro (Model 4). The conditional indirect association was tested using Model 14, with physical activity as a moderator. Variables were mean-centered, and indirect effects were estimated using 5,000 bootstrap samples with 95% bias-corrected confidence intervals (76).

When significant interactions were identified, simple-slope analyses were conducted at the observed 25th, 50th, and 75th percentile scores of physical activity. Interaction effect sizes were reported using ΔR^2^ and Cohen’s f^2^ (77). Additional analyses examined whether physical activity moderated the direct association between childhood maltreatment and NSSI and whether nonlinear effects of physical activity were present.

Because all variables were measured simultaneously, findings were interpreted as cross-sectional associations rather than causal relationships. Sensitivity analyses using the PHQ-8 were conducted to test robustness. Because the NSSI behavior score contained a substantial proportion of zero values and showed overdispersion, a zero-inflated negative binomial regression was conducted as an additional sensitivity analysis. The model included childhood maltreatment, depressive symptoms, physical activity, the depressive symptoms × physical activity interaction, and the same demographic and socioeconomic covariates used in the primary conditional-process analysis. Depressive symptoms and physical activity were mean-centered before constructing the interaction term. The zero-inflation component contained an intercept only. Model fit was compared with the standard negative binomial model using the Akaike information criterion and Bayesian information criterion.

To address equivalent-model ambiguity in the cross-sectional indirect-effect and conditional-process analyses, three alternative directional indirect-effect models and one alternative conditional-process model were estimated using the same covariates and 5,000 bootstrap samples. These analyses were treated as sensitivity tests and were not interpreted as evidence of temporal or causal ordering.

Network analysis was conducted in R to examine relationships at the dimension and symptom levels. The network included 18 nodes and was estimated using EBICglasso with γ = .50. Bridge expected influence, edge-weight accuracy, and node stability were evaluated using bootstrap procedures (78, 79). A sensitivity analysis excluding PHQ-9 Item 9 was also performed.

Province-, school-, and class-level identifiers were not retained in the dataset; therefore, multilevel analyses could not be conducted. This limitation was considered when interpreting the results.

## 3 Results

### 3.1 Common method bias test

A confirmatory factor analysis-based approach was used to evaluate the extent to which the associations among the study variables might be attributable to a single common method factor. The theoretically specified four-factor model, representing childhood maltreatment, depressive symptoms, NSSI behavior, and physical activity, showed substantially better fit than the single-factor model. The four-factor model yielded χ^2^(71) = 1207.983, p <.001, CFI = .948, TLI = .933, RMSEA = .082, and SRMR = .050. Although the RMSEA was slightly above the conventional criterion of.080, the CFI, TLI, and SRMR indicated an acceptable comparative fit.

In contrast, the single-factor model showed poor fit, χ^2^(77) = 8691.862, p <.001, CFI = .603, TLI = .531, RMSEA = .218, and SRMR = .200. The difference between the two models was substantial, Δχ^2^(6) = 7483.880, p <.001. These findings indicated that a single common factor could not adequately account for the covariance among the measures. Nevertheless, the possibility of some residual common method variance could not be completely excluded.

### 3.2 Descriptive statistics and correlation analysis

Descriptive statistics and Pearson correlations for the main study variables are presented in **Table 1**. Physical activity and NSSI behavior showed positively skewed distributions, whereas childhood maltreatment and depressive symptoms were comparatively closer to symmetry. Accordingly, HC3 heteroscedasticity-consistent standard errors and bootstrap confidence intervals were used in the subsequent regression analyses. No variable transformation was applied.

**Table 1:** Descriptive statistics and zero-order correlations among the primary study variables.

Variable	M	SD	Skewness	Kurtosis	1	2	3	4
Childhood maltreatment	41.484	15.843	0.813	0.059	—			
Depressive symptoms	7.979	5.491	0.309	-0.599	0.365***	—		
NSSI behavior	2.339	8.125	4.792	21.753	0.366***	0.531***	—	
Physical activity	12.086	13.511	2.264	7.041	−0.051*	−0.188***	−0.176***	—

N = 2,354. Childhood maltreatment = sum of the 25 clinical CTQ-SF items; depressive symptoms = PHQ-9 total score; NSSI behavior = recoded 12-item behavior total score; physical activity = intensity × (duration − 1) × frequency. *p <.05. ***p <.001.

Childhood maltreatment was positively correlated with depressive symptoms (r = .365, p <.001) and NSSI behavior (r = .366, p <.001). Depressive symptoms were positively correlated with NSSI behavior (r = .531, p <.001). Physical activity was negatively correlated with childhood maltreatment (r = −.051, p = .013), depressive symptoms (r = −.188, p <.001), and NSSI behavior (r = −.176, p <.001).

### 3.3 Test of the cross-sectional indirect effect

The cross-sectional indirect association of childhood maltreatment with NSSI behavior through depressive symptoms was examined after adjustment for grade, sex, ethnicity, family residence, left-behind status, boarding status, only-child status, paternal education, maternal education, monthly household income, and family type. The adjusted analyses were based on 2,353 complete cases.

As shown in **Table 2**, childhood maltreatment was positively associated with depressive symptoms, B = .126, HC3 SE = .008, t = 16.453, p <.001, 95% CI [.111, .140]. The total association between childhood maltreatment and NSSI behavior was also significant, B = .188, HC3 SE = .013, t = 14.868, p <.001, 95% CI [.163, .212].

**Table 2:** Covariate-adjusted regression models for the cross-sectional indirect association.

Model	Outcome	Predictor	B	HC3 SE	t	95% CI	R^2^
1	Depressive symptoms	Childhood maltreatment	0.126	0.008	16.453***	[.111, .140]	0.144
2	NSSI behavior	Childhood maltreatment	0.188	0.013	14.868***	[.163, .212]	0.149
3	NSSI behavior	Childhood maltreatment	0.103	0.011	9.290***	[.082, .125]	0.325
		Depressive symptoms	0.671	0.032	21.121***	[.609, .733]	

N = 2,353. Model 1 is the mediator model (childhood maltreatment → depressive symptoms). Model 2 is the total-association model (childhood maltreatment → NSSI behavior). Model 3 is the outcome model containing both childhood maltreatment and depressive symptoms. All models controlled for grade, sex, ethnicity, family residence, left-behind status, boarding status, only-child status, paternal education, maternal education, monthly household income, and family type. HC3 = heteroscedasticity-consistent standard error. ***p <.001.

After depressive symptoms were included in the outcome model, childhood maltreatment remained positively associated with NSSI behavior, B = .103, HC3 SE = .011, t = 9.290, p <.001, 95% CI [.082, .125]. Depressive symptoms were also positively associated with NSSI behavior, B = .671, HC3 SE = .032, t = 21.121, p <.001, 95% CI [.609, .733].

The product-of-coefficients estimate of the indirect association was.084. The 5,000-sample bootstrap analysis produced an indirect-effect estimate of.084, BootSE = .007, 95% CI [.072, .098]. The confidence interval did not include zero. The indirect association accounted for approximately 44.91% of the total association, whereas the remaining direct association accounted for approximately 55.09%.

These findings supported a statistically significant cross-sectional indirect association through depressive symptoms. However, because childhood maltreatment, depressive symptoms, and NSSI were assessed simultaneously, these results did not establish temporal precedence or causal mediation. The decomposition of the total, direct, and indirect associations is summarized in **Table 3**, and the adjusted indirect-effect model is shown in **Figure 2**.

**Table 3:** Decomposition of the total, direct, and indirect associations.

Effect	Estimate	SE/BootSE	95% CI	Proportion of total association
Total association	0.188	0.013	[.163, .212]	100.00%
Direct association	0.103	0.011	[.082, .125]	55.09%
Indirect association through depressive symptoms	0.084	0.007	[.072, .098]	44.91%

N = 2,353. HC3 standard errors and confidence intervals are reported for the total and direct associations. The indirect-association BootSE and confidence interval were estimated using 5,000 bootstrap samples. Proportions were calculated using the unrounded effect estimates; minor discrepancies in the displayed totals are due to rounding.

**Figure 2:**
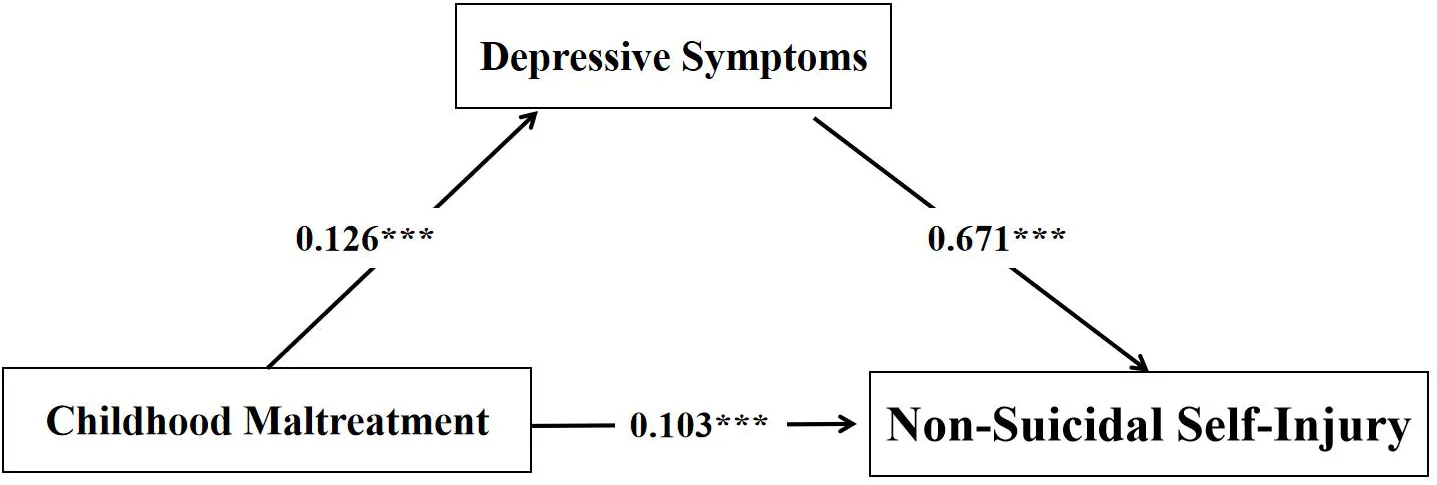
Cross-sectional indirect-effect model. Values are adjusted unstandardized coefficients. NSSI refers to the corrected 12-item behavior total. The indirect association was.084, 95% bootstrap CI [.072, .098]. N = 2,353. ***p <.001.

### 3.4 Test of the conditional indirect effect

The conditional process model examined whether physical activity moderated the association between depressive symptoms and NSSI behavior. As shown in **Table 4**, childhood maltreatment remained positively associated with NSSI behavior, B = .092, HC3 SE = .011, t = 8.526, p <.001. Depressive symptoms were positively associated with NSSI behavior, B = .592, HC3 SE = .033, t = 17.941, p <.001, whereas physical activity was negatively associated with NSSI behavior, B = −.068, HC3 SE = .011, t = −6.366, p <.001.

**Table 4:** Covariate-adjusted regression model for the conditional process analysis.

Outcome	Predictor	B	HC3 SE	t	95% CI	R^2^
NSSI behavior	Childhood maltreatment	.092	.011	8.526***	[.071, .113]	.344
	Depressive symptoms	.592	.033	17.941***	[.528, .657]	
	Physical activity	−.068	.011	−6.366***	[−.088, −.047]	
	Depressive symptoms × physical activity	−.015	.003	−5.845***	[−.021, −.010]	

N = 2,353. Depressive symptoms and physical activity were mean-centered before construction of the interaction term. The interaction accounted for ΔR^2^ = .013, corresponding to Cohen’s f^2^ = .019, indicating a very small effect below the conventional.02 threshold. The model controlled for the same demographic and socioeconomic variables as **Table 2**. HC3 = heteroscedasticity-consistent standard error. ***p <.001.

The depressive symptoms × physical activity interaction was statistically significant and negative, B = −.015, HC3 SE = .003, t = −5.845, p <.001, 95% CI [−.021, −.010]. However, the interaction explained only approximately 1.3% of additional variance in NSSI behavior (ΔR^2^ = .013). Cohen’s f^2^ was.019, which was below the conventional.02 threshold for a small effect and therefore indicated a very small effect with limited practical significance.

The index of moderated indirect association was −.00194, BootSE = .00034, 95% CI [−.00263, −.00128]. The conditional indirect association decreased as physical activity increased. At the 25th percentile of physical activity, the conditional indirect association was.092, 95% CI [.079, .106]. At the median, it was.082, 95% CI [.070, .095]. At the 75th percentile, it decreased to.063, 95% CI [.051, .076]. These conditional indirect associations are summarized in **Table 5**.

**Table 5:** Conditional indirect associations and index of moderated indirect association.

Effect/physical activity level	Observed PA score	Estimate	BootSE	Bootstrap 95% CI
Index of moderated indirect association	—	−.00194	.00034	[−.00263, −.00128]
Conditional indirect association: 25th percentile	3	.092	.007	[.079, .106]
Conditional indirect association: 50th percentile	8	.082	.006	[.070, .095]
Conditional indirect association: 75th percentile	18	.063	.006	[.051, .076]

N = 2,353. The indirect association is childhood maltreatment → depressive symptoms → NSSI behavior. Because physical activity was positively skewed, conditional estimates were probed at observed percentile scores rather than at the mean ± 1 SD. Confidence intervals were estimated using 5,000 bootstrap samples.

An additional model was used to test whether physical activity also moderated the direct association between childhood maltreatment and NSSI. The childhood maltreatment × physical activity interaction was not significant, B = .00047, HC3 SE = .00087, t = .545, p = .586, 95% CI [−.00123, .00218]. Thus, physical activity did not significantly moderate the direct association between childhood maltreatment and NSSI in the present model. However, this nonsignificant interaction does not establish the temporal or theoretical placement of physical activity within the proposed model.

The quadratic physical activity term was also not significant, B = .00010, HC3 SE = .00040, t = .257, p = .797, 95% CI [−.00067, .00088]. Therefore, no evidence of a nonlinear association between physical activity and NSSI was identified within the observed score range. The adjusted conditional-process model is shown in **Figure 3**.

**Figure 3:**
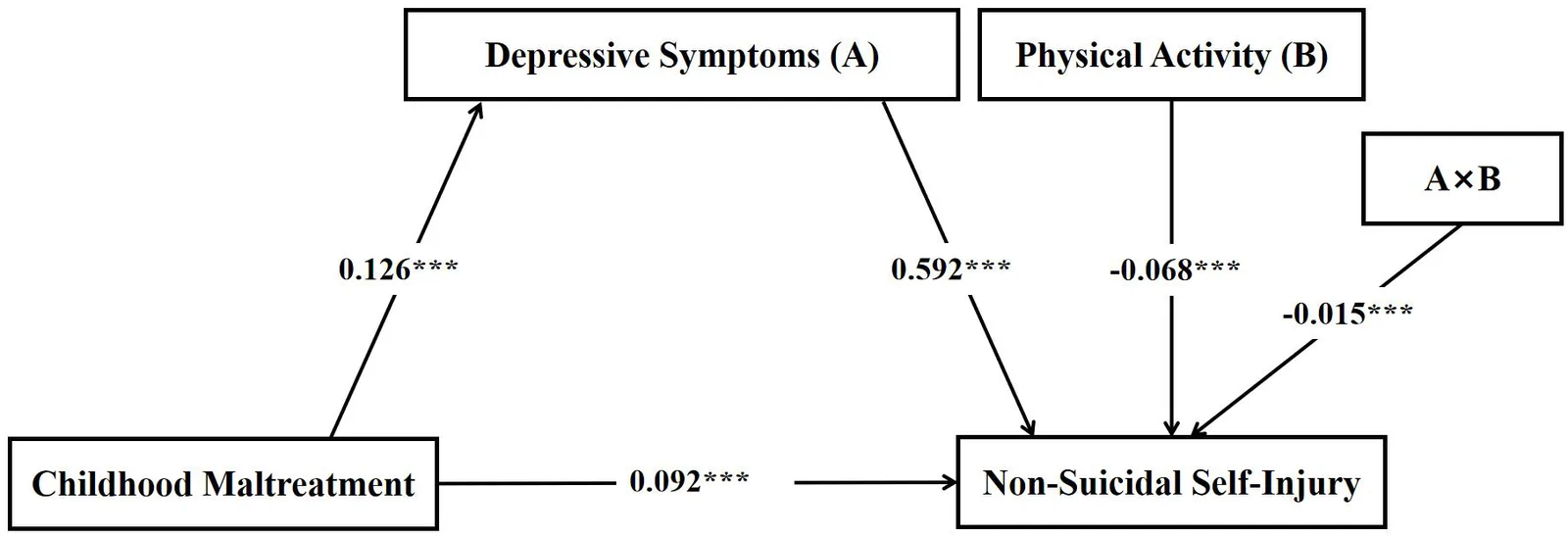
Cross-sectional conditional process model. Values are adjusted unstandardized coefficients; continuous predictors were mean-centered. The index of moderated indirect association was −.00194, 95% bootstrap CI [−.00263, −.00128]. N = 2,353. ***p <.001.

### 3.5 Sensitivity analysis using the PHQ-8 score

Sensitivity analyses were conducted after excluding PHQ-9 Item 9. Childhood maltreatment remained positively associated with PHQ-8 depressive symptoms, B = .114, HC3 SE = .007, t = 17.267, p <.001, 95% CI [.101, .127]. PHQ-8 depressive symptoms remained positively associated with NSSI behavior, B = .760, HC3 SE = .036, t = 20.848, p <.001, 95% CI [.688, .831].

The indirect association through PHQ-8 depressive symptoms was.087, BootSE = .007, 95% CI [.074, .100]. In the conditional process model, PHQ-8 depressive symptoms were positively associated with NSSI behavior, B = .674, HC3 SE = .038, t = 17.940, p <.001, whereas physical activity was negatively associated with NSSI behavior, B = −.068, HC3 SE = .011, t = −6.350, p <.001. The PHQ-8 × physical activity interaction remained significant, B = −.017, HC3 SE = .003, t = −5.944, p <.001, 95% CI [−.023, −.012].

The index of moderated indirect association was −.00199, BootSE = .00035, 95% CI [−.00270, −.00133]. These findings indicated that the main results were not attributable solely to conceptual overlap between the PHQ-9 self-harm item and the NSSI outcome.

### 3.6 Simple slope analysis

Given the positive skewness of physical activity, simple slopes were estimated at its observed 25th, 50th, and 75th percentile scores. As shown in **Table 6** and **Figure 4**, depressive symptoms were positively associated with NSSI behavior at all three levels of physical activity. However, the magnitude of this association decreased as physical activity increased.

**Table 6:** Simple slopes of depressive symptoms predicting NSSI behavior at observed levels of physical activity.

Physical activity level	Observed PA score	Centered PA value	B	HC3 SE	t	95% CI
25th percentile	3	−9.086	.732	.035	21.044***	[.664, .800]
50th percentile	8	−4.086	.655	.032	20.689***	[.593, .717]
75th percentile	18	5.914	.502	.040	12.440***	[.423, .581]

N = 2,353. B represents the conditional association between depressive symptoms and NSSI behavior at each observed physical activity score. HC3 = heteroscedasticity-consistent standard error. ***p <.001.

**Figure 4:**
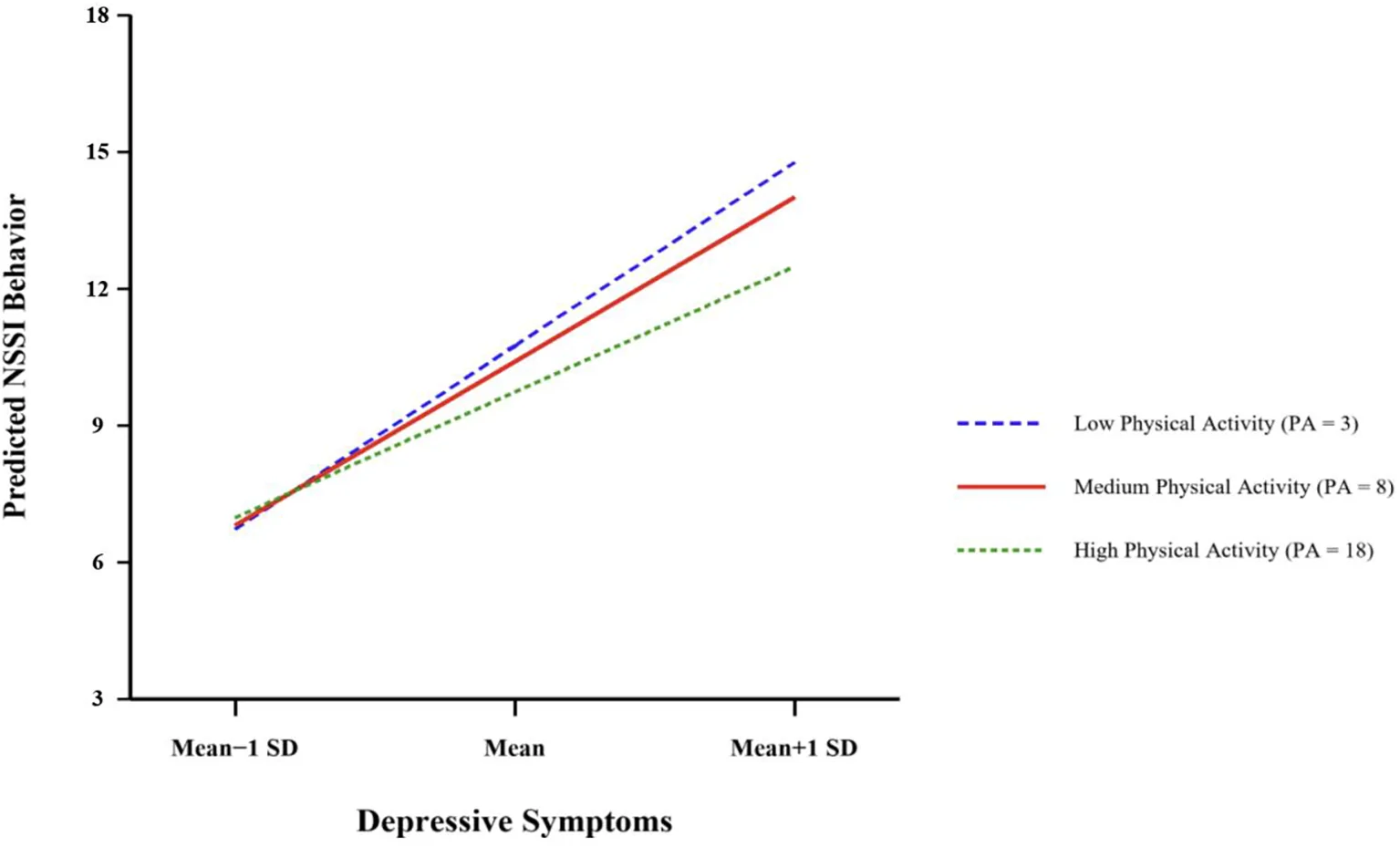
Simple slope analysis. PA = 3, 8, and 18 correspond to the observed 25th, 50th, and 75th percentiles. The adjusted slopes were B = .732, .655, and.502, respectively; all p <.001. N = 2,353.

At the 25th percentile of physical activity, the association between depressive symptoms and NSSI behavior was B = .732, HC3 SE = .035, t = 21.044, p <.001, 95% CI [.664, .800]. At the median, the association was B = .655, HC3 SE = .032, t = 20.689, p <.001, 95% CI [.593, .717]. At the 75th percentile, the association decreased to B = .502, HC3 SE = .040, t = 12.440, p <.001, 95% CI [.423, .581].

These results indicated that the positive association between depressive symptoms and NSSI behavior was slightly weaker at higher observed levels of physical activity. However, the differences between the slopes were small, and the interaction accounted for only 1.3% of additional variance. Therefore, this pattern should not be interpreted as evidence of a practically or clinically meaningful buffering or protective effect.

### 3.7 Network structure and between-community connectivity

A regularized partial-correlation network containing 18 nodes was estimated. The nodes comprised five childhood maltreatment dimensions, nine individual depressive symptoms, one NSSI behavior node, and three physical activity components. The NSSI function dimension was not included. The final EBICglasso network retained 99 of the 153 possible edges.

The strongest edges were predominantly within the same community. The strongest within-community edge was observed between emotional abuse and physical abuse (edge weight = .517), followed by physical activity intensity and duration (edge weight = .415), physical abuse and physical neglect (edge weight = .399), anhedonia and depressed mood (edge weight = .361), and physical activity duration and frequency (edge weight = .332).

Several notable between-community connections were identified. The strongest depressive symptom–NSSI edge connected concentration difficulty with NSSI behavior (edge weight = .108), followed by sleep problems and NSSI behavior (edge weight = .100), fatigue and NSSI behavior (edge weight = .086), and psychomotor change and NSSI behavior (edge weight = .070). Emotional neglect was connected with sleep problems (edge weight = .107) and NSSI behavior (edge weight = .092). Physical neglect was connected with appetite change (edge weight = .092).

Physical activity showed relatively weak cross-community connections. NSSI behavior was negatively connected with physical activity intensity (edge weight = −.076) and frequency (edge weight = −.047), whereas fatigue was negatively connected with physical activity duration (edge weight = −.057). The absolute magnitudes of these physical activity edges were smaller than those of the dominant within-community edges. Thus, after controlling for the remaining nodes, physical activity showed only weak unique associations with NSSI behavior and fatigue. The estimated network is shown in **Figure 5**, and the strongest between-community edges are summarized in **Figure 6**.

**Figure 5:**
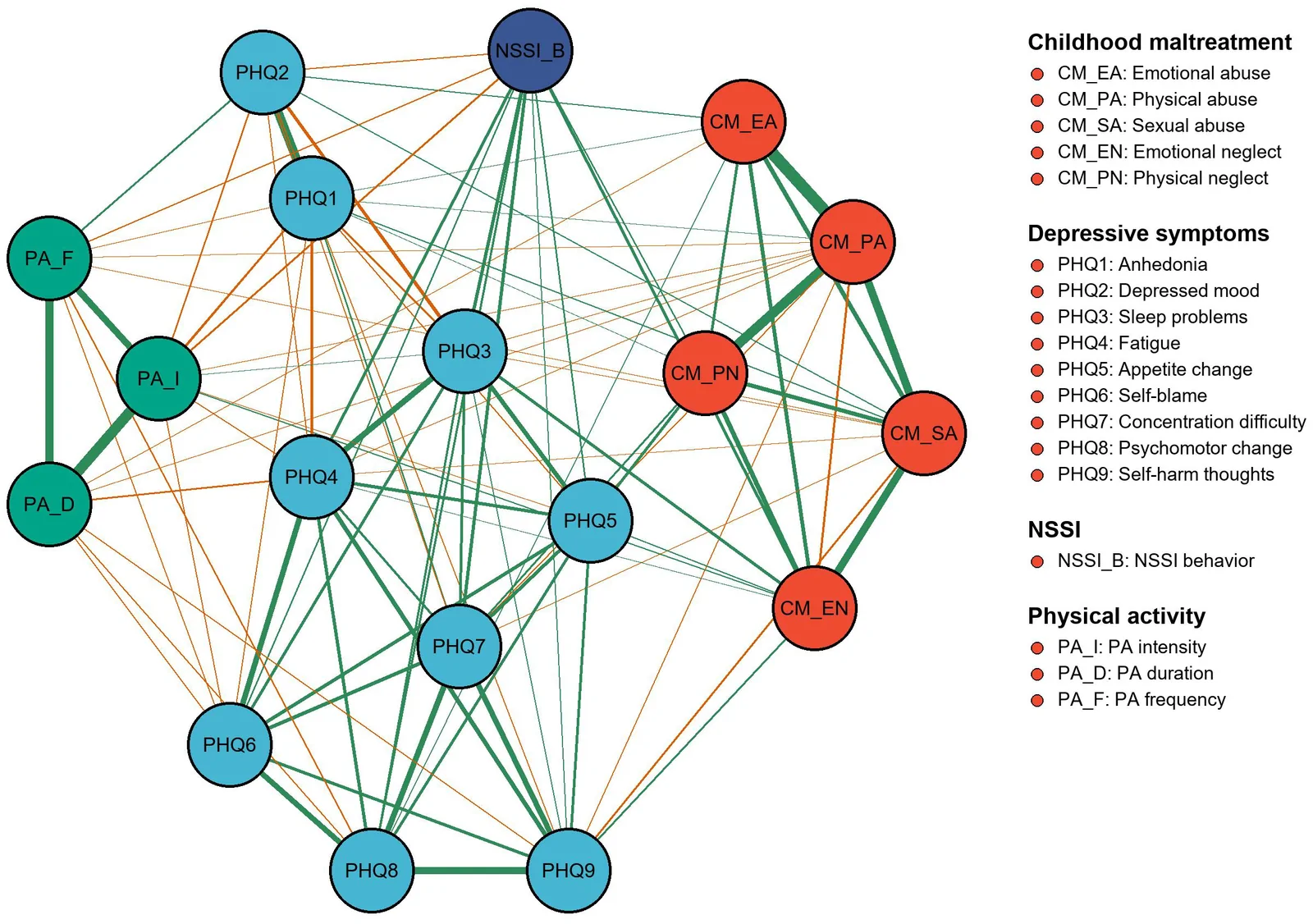
Regularized partial correlation network of childhood maltreatment, depressive symptoms, NSSI behavior, and physical activity. Green edges indicate positive associations, orange edges indicate negative associations, and thicker edges indicate stronger absolute regularized partial correlations. The corrected network contained 18 nodes and 99 nonzero edges; the NSSI function dimension was excluded. N = 2,354.

**Figure 6:**
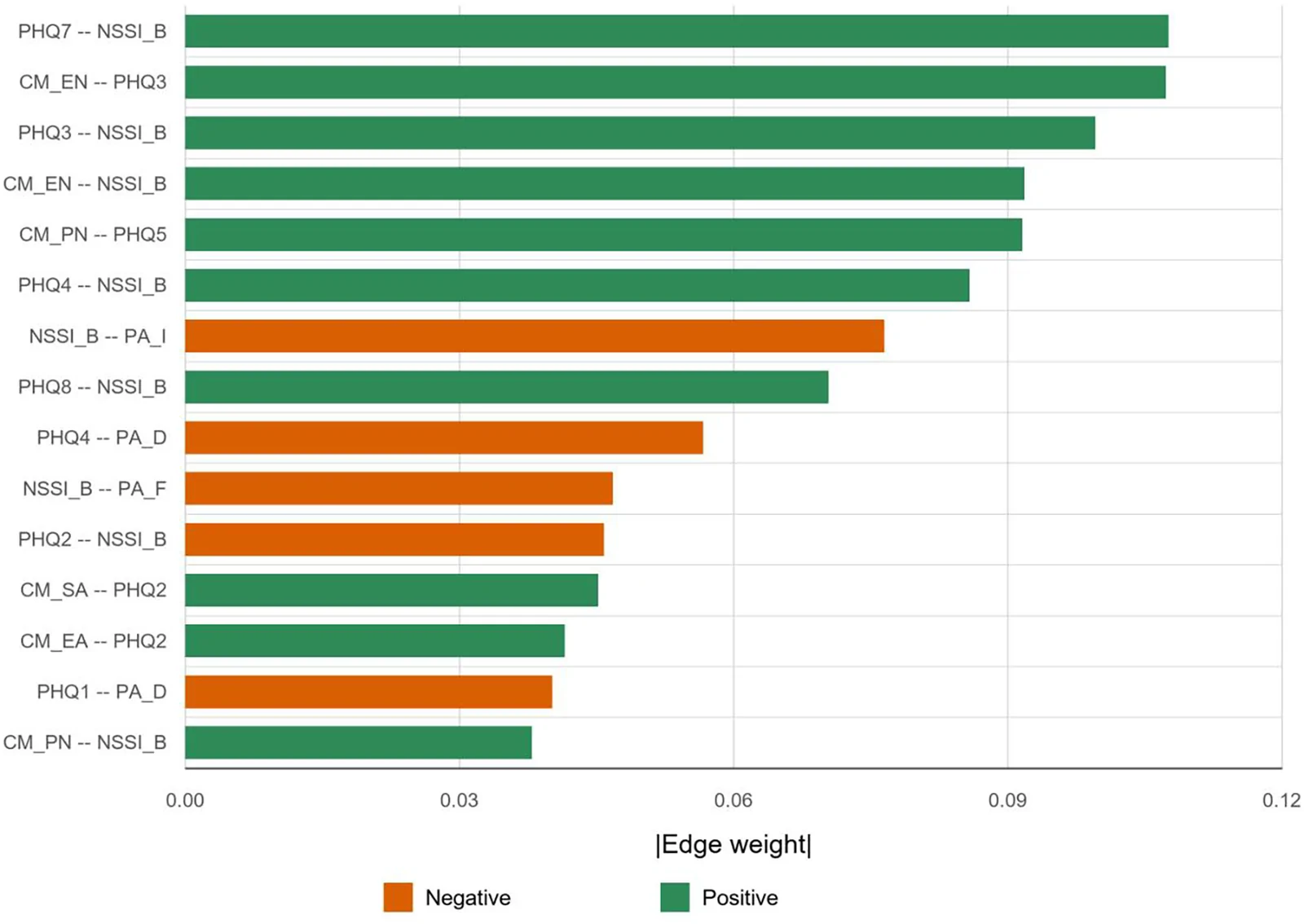
Strongest between-community edges in the network. Bars show absolute edge weights and are ordered from largest to smallest. Green indicates a positive edge and orange indicates a negative edge. N = 2,354.

### 3.8 Bridge centrality analysis

Bridge expected influence was calculated to identify nodes connecting the four communities. NSSI behavior, which constituted a single-node community, showed high cross-community connectivity, with a bridge expected-influence value of.401. Among nodes belonging to the multi-node communities, emotional neglect showed the highest positive bridge expected influence (bridge EI = .317), followed by sleep problems (bridge EI = .193) and physical neglect (bridge EI = .182). Appetite change (bridge EI = .141) and concentration difficulty (bridge EI = .132) also showed relatively high positive bridge values.

The bridge expected-influence values of physical activity intensity and duration were −.149 and −.148, respectively, reflecting their predominantly negative connections with nodes outside the physical activity community. Physical activity frequency showed a smaller bridge expected influence of −.088. Thus, emotional neglect, sleep problems, and physical neglect were the most prominent positive bridge nodes among the multi-node communities, whereas physical activity showed relatively weak and predominantly negative cross-community associations. Because NSSI behavior constituted a single-node community, its bridge expected-influence value was not interpreted as directly comparable with values from the multi-node communities. Bridge expected-influence values are shown in **Figure 7**.

**Figure 7:**
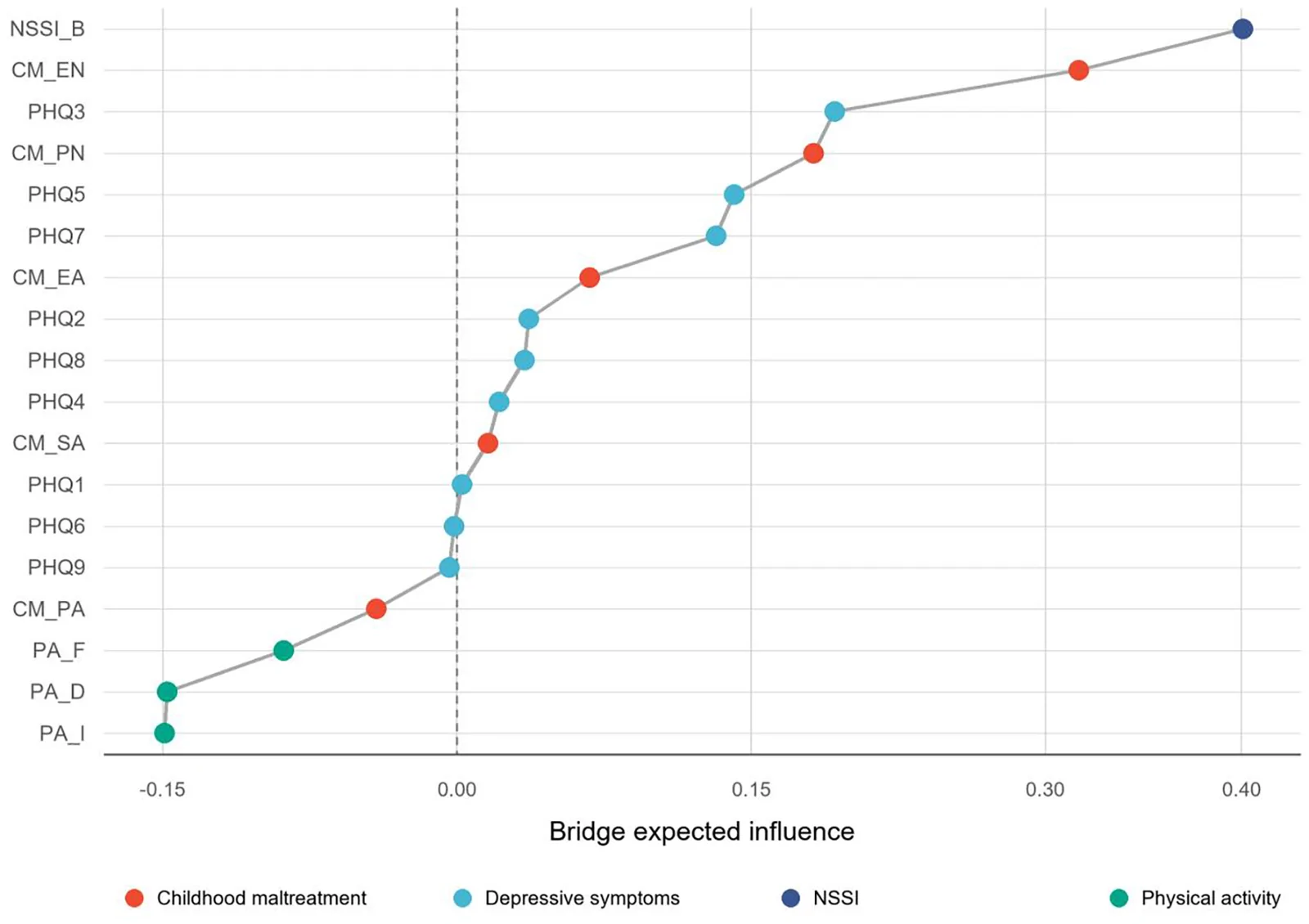
Bridge expected influence of nodes in the network. Values are signed sums of a node’s edges to other communities. The case-dropping correlation-stability coefficient was CS = .70. N = 2,354.

### 3.9 Network accuracy and stability

Nonparametric bootstrap analyses showed that the strongest within-community edges were estimated with comparatively narrow confidence intervals. For example, the 95% bootstrap confidence interval was [.480, .548] for the emotional abuse–physical abuse edge and [.380, .444] for the physical activity intensity–duration edge. Smaller edges were interpreted cautiously because edge-weight rank differences are less reliable when bootstrap confidence intervals overlap.

The case-dropping bootstrap analysis indicated that bridge expected influence was stable, with a correlation-stability coefficient of.70, exceeding the recommended value of.50. In the network sensitivity analysis excluding PHQ-9 Item 9, the correlation between bridge expected-influence values in the primary and sensitivity networks was.999. The single NSSI node continued to show high cross-community connectivity, while emotional neglect, sleep problems, and physical neglect remained the leading positive bridge nodes among the multi-node communities. These results supported the stability of the principal network findings and indicated that they were not driven by the PHQ-9 self-harm item.

### 3.10 Zero-inflated negative binomial sensitivity analysis

Among the 2,353 participants included in the adjusted analyses, 1,010 (42.92%) had an NSSI behavior score of zero. The NSSI outcome also showed substantial overdispersion. The zero-inflated negative binomial model showed a better fit than the standard negative binomial model, with lower AIC and BIC values (AIC = 11237.5 vs. 11246.8; BIC = 11358.5 vs. 11362.1). The dispersion parameter was statistically significant, α = .420, SE = .050, p <.001.

In the count component of the zero-inflated negative binomial model, childhood maltreatment was positively associated with the expected NSSI behavior score, IRR = 1.006, 95% CI [1.003, 1.009], p <.001. Depressive symptoms were also positively associated with the expected NSSI behavior score, IRR = 1.074, 95% CI [1.061, 1.086], p <.001, whereas physical activity was negatively associated with the expected NSSI behavior score, IRR = .991, 95% CI [.987, .995], p <.001. The depressive symptoms × physical activity interaction remained statistically significant, IRR = .998, 95% CI [.996, .999], p <.001. Thus, the directions and statistical significance of the principal regression findings were retained when zero inflation and overdispersion were explicitly modeled. Detailed coefficients are presented in **Supplementary Table 2**.

### 3.11 Alternative directional model analyses

Alternative directional analyses were conducted to examine whether the hypothesized ordering was uniquely supported by the cross-sectional data. In the childhood maltreatment → NSSI behavior → depressive symptoms model, the indirect association was statistically significant, effect = .058, BootSE = .005, 95% bootstrap CI [.049, .068]. In the NSSI behavior → depressive symptoms → childhood maltreatment model, the indirect association was also statistically significant, effect = .241, BootSE = .030, 95% bootstrap CI [.183, .299]. The NSSI behavior → depressive symptoms → physical activity model similarly produced a statistically significant negative indirect association, effect = −.118, BootSE = .020, 95% bootstrap CI [−.159, −.080].

An alternative conditional-process model was also estimated in which childhood maltreatment was associated with depressive symptoms through NSSI behavior and physical activity moderated the NSSI behavior–depressive symptoms association. The interaction was statistically significant, B = −.0080, HC3 SE = .0010, p <.001, and the index of moderated indirect association was −.00150, BootSE = .00022, 95% bootstrap CI [−.00197, −.00110].

These findings indicated that the cross-sectional data did not uniquely distinguish the hypothesized ordering from alternative directional configurations. The hypothesized model was therefore retained as an *a priori*, theory-guided representation rather than as an empirically established temporal sequence. Detailed results of the alternative indirect-effect and conditional-process models are presented in **Supplementary Tables 3**, **4**.

## 4 Discussion

The present cross-sectional study examined the associations among childhood maltreatment, depressive symptoms, physical activity, and NSSI behavior in Chinese adolescents using complementary variable-level and network approaches. Childhood maltreatment was positively associated with depressive symptoms and NSSI behavior, and depressive symptoms showed a statistically significant indirect association between childhood maltreatment and NSSI. A statistically significant interaction between depressive symptoms and physical activity was also identified, but it explained only 1.3% of additional variance and was very small in magnitude. Network analysis further identified emotional neglect, sleep problems, and physical neglect as prominent positive bridge nodes, while the single NSSI node showed high cross-community connectivity. Taken together, the findings primarily highlight the associations of adverse caregiving experiences and depressive distress with NSSI, whereas the practical contribution of physical activity was minimal. Because all variables were assessed simultaneously, the findings should be interpreted as cross-sectional associations rather than as evidence of developmental, causal, protective, or clinically meaningful effects.

First, childhood maltreatment was positively associated with adolescent NSSI behavior. This finding is consistent with previous research indicating that adverse caregiving experiences constitute an important developmental correlate of adolescent self-injury (64, 80). Adolescents exposed to emotional abuse, neglect, or chronically unsafe family environments may experience sustained emotional distress, low perceived support, heightened threat sensitivity, and difficulty developing adaptive emotion-regulation strategies (81–84). Under these conditions, NSSI may be adopted as a maladaptive strategy for reducing intense affect, interrupting emotional numbness, or regaining a temporary sense of control. Nevertheless, the present data cannot determine whether childhood maltreatment preceded the emergence of NSSI in this sample or whether additional unmeasured family and individual characteristics contributed to both variables.

Second, childhood maltreatment was positively associated with depressive symptoms. Adolescents exposed to maltreatment and emotionally unresponsive family environments may be more likely to report negative affect, low self-worth, hopelessness, fatigue, sleep disturbance, and cognitive-emotional withdrawal (85–88). From a developmental and social-psychological perspective, depressive symptoms should not be viewed exclusively as internal individual problems detached from interpersonal context. Instead, they may also reflect psychological distress occurring in the context of prolonged relational strain, insufficient emotional responsiveness, and limited support resources (89–92). The current findings are therefore consistent with the view that depressive symptoms represent an important proximal correlate of adverse childhood experiences.

Depressive symptoms were also positively associated with NSSI behavior and statistically accounted for part of the association between childhood maltreatment and NSSI. This pattern is consistent with the hypothesized possibility that adolescents exposed to maltreatment may experience greater depressive distress, which may, in turn, be associated with a greater frequency of NSSI behavior (93, 94). Negative self-evaluation, hopelessness, emotional pain, and reduced coping resources may make self-injury more likely to be considered or used as a maladaptive emotion-regulation strategy.

Importantly, this indirect effect should not be interpreted as evidence that childhood maltreatment caused depressive symptoms and that depressive symptoms subsequently caused NSSI. Cross-sectional indirect-effect models do not establish the temporal ordering required for causal mediation. Reverse and bidirectional relationships are also plausible. For example, recurrent NSSI may contribute to subsequent depressive symptoms through shame, interpersonal conflict, or functional impairment, while depression and NSSI may mutually reinforce one another over time (95, 96). Thus, the present result identifies a theoretically meaningful pattern of shared associations, but longitudinal measurements are required to determine whether the proposed temporal sequence is supported.

The alternative directional analyses further illustrated this equivalent-model problem. Significant indirect associations were also obtained when NSSI behavior was positioned before depressive symptoms and when depressive symptoms were positioned before childhood maltreatment or physical activity. The alternative conditional-process model likewise yielded a significant index of moderated indirect association. These findings do not invalidate the hypothesized model, but they indicate that the present cross-sectional data cannot establish its unique temporal ordering or empirical superiority over alternative configurations. The hypothesized model was therefore retained as an *a priori*, theory-guided representation because childhood maltreatment was conceptualized as a distal developmental exposure, whereas depressive symptoms and NSSI were conceptualized as more proximal concurrent correlates.

The PHQ-8 sensitivity analysis represents an important methodological strength. PHQ-9 Item 9 assesses thoughts of death or self-harm and may overlap conceptually with an NSSI outcome, even though suicidal thoughts and NSSI are not equivalent constructs (97, 98). After Item 9 was removed, the indirect association, depressive symptoms × physical activity interaction, and conditional indirect association all remained statistically significant. In addition, bridge expected-influence values in the PHQ-9 and PHQ-8 networks were nearly identical (r = .999). These results indicate that the principal findings were not driven solely by the inclusion of a self-harm-related item in the depressive symptom measure.

The zero-inflated negative binomial sensitivity analysis further supported the robustness of the principal regression findings. Childhood maltreatment, depressive symptoms, physical activity, and the depressive symptoms × physical activity interaction retained the same directions and remained statistically significant after the zero concentration and overdispersion of the NSSI outcome were explicitly modeled. Nevertheless, the interaction remained very small and should not be interpreted as evidence of a practically or clinically meaningful protective effect.

The network analysis provided additional dimension- and symptom-level information. Emotional neglect was connected with sleep problems and NSSI behavior, while physical neglect was connected with appetite change. Concentration difficulty, sleep problems, fatigue, and psychomotor change showed positive conditional connections with NSSI behavior. These findings indicate that associations between childhood maltreatment, depressive symptoms, and NSSI were not distributed uniformly across all dimensions and symptoms. Emotional neglect and physical neglect may be particularly relevant caregiving deficits, whereas sleep problems, fatigue, and concentration difficulty may reflect disturbances in emotional, physiological, and cognitive functioning that co-occur with NSSI (99, 100).

The bridge centrality results further supported the importance of emotional neglect and sleep problems. NSSI behavior showed high cross-community connectivity; however, because it constituted a single-node community, its bridge expected-influence value was not interpreted as directly comparable with values from the multi-node communities. Among the multi-node communities, emotional neglect showed the highest positive bridge expected influence, followed by sleep problems and physical neglect. Appetite change and concentration difficulty also showed relatively high bridge values. These nodes may represent candidate points for further longitudinal investigation. However, bridge centrality does not demonstrate that intervening on a node will cause changes in other nodes. Therefore, the bridge findings should be regarded as hypothesis-generating evidence rather than as direct evidence of intervention targets (101, 102).

The present study identified a statistically significant interaction between depressive symptoms and physical activity in relation to NSSI behavior. The positive association between depressive symptoms and NSSI was slightly weaker at higher observed levels of physical activity, whereas the childhood maltreatment × physical activity interaction was not significant. However, the magnitude of the depressive symptoms × physical activity interaction was very small. It explained only approximately 1.3% of additional variance and had a Cohen’s f^2^ of.019, which was below the conventional.02 threshold for a small effect. Given the large sample size, the analysis had substantial statistical power to detect an effect of limited magnitude. Statistical significance should therefore not be equated with practical or clinical importance.

Accordingly, the present findings do not support describing physical activity as a meaningful buffering or protective factor against NSSI. At most, physical activity was associated with a slight variation in the strength of the concurrent depressive symptoms–NSSI relationship. Cognitive-behavioral, affective, physiological, and social mechanisms may theoretically contribute to this association, but none of these mechanisms was directly measured in the present study. The physical activity result should therefore be interpreted with considerable caution and regarded as a weak, non-specific cross-sectional correlate rather than as a central explanatory, preventive, or treatment mechanism. Longitudinal studies using objective and detailed measures of physical activity are required to determine whether this small statistical interaction is replicable or clinically relevant.

The physical activity-related network findings were substantially weaker than the regression-based interaction. Physical activity intensity and frequency showed negative conditional associations with NSSI behavior, and physical activity duration was negatively connected with fatigue. However, these cross-community edges were considerably weaker than the dominant within-community edges. Because the network estimated regularized partial correlations after controlling for all other nodes, the weak edges indicate that physical activity had little unique direct association with NSSI or individual depressive symptoms after shared variance was removed.

The divergence should therefore be interpreted substantively rather than solely as a consequence of methodological differences. The regression model tested whether the association between aggregate depressive symptoms and aggregate NSSI varied across levels of aggregate physical activity, whereas the network did not estimate an interaction term. Part of the broader association involving physical activity may reflect variance shared with depressive and somatic symptoms, including fatigue, rather than a direct and specific association with NSSI. Taken together, the results are more consistent with physical activity functioning as a general, non-specific correlate of psychological and somatic well-being than as a symptom-specific or clinically meaningful buffer against NSSI.

From a practical perspective, the findings suggest that adolescent NSSI prevention should consider developmental adversity, current depressive distress, specific symptom patterns, and available behavioral resources. Adolescents reporting childhood maltreatment may benefit from trauma-informed assessment and careful screening for depressive symptoms and NSSI behavior (94, 99, 103). Sleep problems, fatigue, appetite change, and concentration difficulty may provide useful clinical information, although their bridge status alone does not establish them as causal treatment targets.

For adolescents already reporting depressive symptoms or NSSI, intervention should prioritize comprehensive mental-health assessment, safety planning, evidence-based psychological support, and development of alternative emotion-regulation strategies (104–107). Schools and families may continue to provide accessible and developmentally appropriate opportunities for voluntary physical activity as part of general health promotion. However, the present findings do not demonstrate that increasing physical activity would produce a clinically meaningful reduction in NSSI. Physical activity should not substitute for professional assessment, safety planning, or evidence-based treatment, particularly for adolescents with recurrent NSSI or severe depressive symptoms.

### 4.1 Limitations and future directions

Several limitations should be acknowledged. First, the cross-sectional design precludes conclusions about temporal ordering or causality. The estimated indirect and conditional indirect associations may differ if depressive symptoms, physical activity, and NSSI are assessed prospectively, and reverse or reciprocal associations remain possible. The significant alternative directional models further indicated that the hypothesized ordering could not be uniquely distinguished from other statistically plausible configurations. Future studies should use multiwave longitudinal designs, ideally with at least three assessment points, to test temporal precedence and within-person change.

Second, convenience sampling may have introduced selection bias. Although participants were recruited from 30 schools across six provinces and represented varied school and residential settings, the sample was not nationally representative, and adolescents from rural family residences were overrepresented. Province, school, and class identifiers and participant-level school type were unavailable; therefore, cluster-robust or multilevel corrections could not be performed. Future research should retain anonymized cluster identifiers, use stratified probability sampling where feasible, and incorporate school-level characteristics into multilevel models.

Third, all principal variables were assessed using retrospective or concurrent self-report measures. Childhood maltreatment reports may be affected by recall processes, while NSSI and depressive symptoms may be underreported because of stigma or social desirability. The brief PARS-3 did not provide objective or detailed information about physical activity, and the PHQ-9 is a screening measure rather than a diagnostic interview. In addition, the NSSI behavior score captured frequency but not medical severity, tissue damage, situational context, suicidal intent assessment, or NSSI functions. Future studies should combine self-report measures with clinical interviews, multi-informant assessments, accelerometer-based monitoring, and separate assessments of NSSI frequency, severity, functions, and clinical course.

Finally, the network analysis was cross-sectional and data-driven. Regularization may shrink meaningful associations, and bridge expected influence may vary across samples and measurement choices. In addition, because NSSI behavior constituted a single-node community, its bridge expected-influence value primarily reflected its total connectivity with the other communities and was not directly comparable with bridge values from the multi-node communities. Accordingly, the identified bridge nodes should not be interpreted as causal pathways or definitive intervention targets. Replication in independent samples, longitudinal network models, and experimental or intervention studies are needed to determine whether changes in the identified nodes precede changes in NSSI. Future research should also examine contextual factors such as family support, peer relationships, school climate, and access to mental-health services.

## 5 Conclusion

The present cross-sectional study found that childhood maltreatment was positively associated with depressive symptoms and NSSI behavior among Chinese adolescents. Depressive symptoms statistically accounted for part of the association between childhood maltreatment and NSSI. Physical activity showed a statistically significant but very small interaction with depressive symptoms, explaining only 1.3% of additional variance in NSSI behavior and providing limited evidence of practical or clinical relevance. The principal regression findings remained stable after removal of the PHQ-9 self-harm item and under the zero-inflated negative binomial specification, although the alternative directional models indicated that the hypothesized variable ordering was not unique.

Network analysis identified emotional neglect, sleep problems, and physical neglect as prominent positive bridge nodes, while the single NSSI node showed high cross-community connectivity; physical activity showed weak and predominantly negative cross-community connections. Methodologically, the integration of conditional-process and network analyses provided complementary variable- and symptom-level evidence. The findings primarily underscore the importance of considering developmental adversity and depressive distress in NSSI assessment, while the physical activity findings should be interpreted with considerable caution. Longitudinal and multi-method research is required before causal, protective, preventive, or treatment conclusions can be drawn.

## Data Availability

The raw data supporting the conclusions of this article will be made available by the authors, without undue reservation.

## Supplementary material

Supplementary Table 1 Demographic and socioeconomic characteristics of the participants.

Supplementary Table 2 Count component of the zero-inflated negative binomial regression predicting the NSSI behavior score.

Supplementary Table 3 Alternative directional indirect-effect models.

Supplementary Table 4 Alternative conditional-process model.
