# Supplementary material for: Childhood maltreatment and adolescent non-suicidal self-injury: cross-sectional conditional process and network analyses of depressive symptoms and physical activity
Source: Front Psychiatry. 2026 Sep 15;17:1899267. doi: 10.3389/fpsyt.2026.1899267 (PMC13621343; doi:10.3389/fpsyt.2026.1899267)
Supplement: Supplementary file 1 [file Table1.docx]

**Supplementary Material**

Childhood Maltreatment and Adolescent Non-Suicidal Self-Injury: Cross-Sectional Conditional Process and Network Analyses of Depressive Symptoms and Physical Activity

Wanchun Xue, Jianhua Zhang, Chang Hong*

## Supplementary Table S1. Descriptive statistics of NSSI behavior

| Indicator | Value |
| --- | --- |
| Sample size | 2,354 |
| Adjusted analysis sample size | 2,353 |
| NSSI mean | 2.339 |
| NSSI standard deviation | 8.125 |
| NSSI skewness | 0.961 |
| NSSI kurtosis | 2.717 |
| NSSI theoretical range | 0–48 |

## Supplementary Table S2. Zero-inflated negative binomial regression predicting the NSSI count outcome

| Predictor | B | SE | IRR | 95% CI for IRR | p |
| --- | --- | --- | --- | --- | --- |
| Childhood maltreatment | 0.0060 | 0.0015 | 1.006 | [1.003, 1.009] | <.001 |
| Depressive symptoms | 0.0710 | 0.0060 | 1.074 | [1.061, 1.086] | <.001 |
| Physical activity | -0.0090 | 0.0020 | 0.991 | [0.987, 0.995] | <.001 |
| Depressive symptoms × physical activity | -0.0025 | 0.00055 | 0.998 | [0.996, 0.999] | <.001 |

## Supplementary Table S3. Alternative directional indirect-effect models

| Model | a | b | c′ | Indirect effect | BootSE | 95% Bootstrap CI |
| --- | --- | --- | --- | --- | --- | --- |
| CM → PHQ → NSSI | 0.126 | 0.671 | 0.103 | 0.084 | 0.007 | [0.072, 0.098] |
| CM → NSSI → PHQ | 0.188 | 0.310 | 0.068 | 0.058 | 0.005 | [0.049, 0.068] |
| NSSI → PHQ → CM | 0.357 | 0.675 | 0.473 | 0.241 | 0.030 | [0.183, 0.299] |
| NSSI → PHQ → PA | 0.357 | -0.331 | -0.170 | -0.118 | 0.020 | [-0.159, -0.080] |

## Supplementary Table S4. Alternative conditional-process models

| Model | Interaction B | HC3 SE | p | Index | BootSE | Bootstrap 95% CI |
| --- | --- | --- | --- | --- | --- | --- |
| CM → NSSI → PHQ; PA moderates NSSI → PHQ | -0.0080 | 0.0010 | <.001 | -0.00150 | 0.00022 | [-0.00197, -0.00110] |
| NSSI → PHQ → CM; PA moderates PHQ → CM | -0.0390 | 0.0060 | <.001 | -0.0139 | 0.0021 | [-0.0183, -0.0099] |
